# Postresectional lung injury in thoracic surgery pre and intraoperative risk factors: a retrospective clinical study of a hundred forty-three cases

**DOI:** 10.1186/1749-8090-5-62

**Published:** 2010-08-17

**Authors:** Serdar Şen, Selda Şen, Ekrem Şentürk, Nilgün Kanlıoğlu Kuman

**Affiliations:** 1 Department of Thoracic Surgery, Medical Faculty, Adnan Menderes University, Aydin, Turkey; 2 Department of Anesthesiology and Reanimation, Medical Faculty, Adnan Menderes University, Aydin, Turkey

## Abstract

**Introduction:**

Acute respiratory dysfunction syndrome (ARDS), defined as acute hypoxemia accompanied by radiographic pulmonary infiltrates without a clearly identifiable cause, is a major cause of morbidity and mortality after pulmonary resection. The aim of the study was to determine the pre and intraoperative factors associated with ARDS after pulmonary resection retrospectively.

**Methods:**

Patients undergoing elective pulmonary resection at Adnan Menderes University Medical Faculty Thoracic Surgery Department from January 2005 to February 2010 were included in this retrospective study. The authors collected data on demographics, relevant co-morbidities, the American Society of Anesthesiologists (ASA) Physical Status classification score, pulmonary function tests, type of operation, duration of surgery and intraoperative fluid administration (fluid therapy and blood products). The primary outcome measure was postoperative ARDS, defined as the need for continuation of mechanical ventilation for greater than 48-hours postoperatively or the need for reinstitution of mechanical ventilation after extubation. Statistical analysis was performed with Fisher exact test for categorical variables and logistic regression analysis for continuous variables.

**Results:**

Of one hundred forty-three pulmonary resection patients, 11 (7.5%) developed postoperative ARDS. Alcohol abuse (p = 0.01, OR = 39.6), ASA score (p = 0.001, OR: 1257.3), resection type (p = 0.032, OR = 28.6) and fresh frozen plasma (FFP)(p = 0.027, OR = 1.4) were the factors found to be statistically significant.

**Conclusion:**

In the light of the current study, lung injury after lung resection has a high mortality. Preoperative and postoperative risk factor were significant predictors of postoperative lung injury.

## Introduction

Major advances in thoracic surgery, intraoperative anesthetic management, and perioperative care over the past 30 years have led to a significant reduction in the postoperative complications of patients undergoing lung resection [1]. Respiratory complications remain the major cause of morbidity and mortality following lung resection. Acute lung injury (ALI) and acute respiratory disease syndrome (ARDS) are responsible for the vast majority of respiratory-related deaths [2].

ARDS formally defined as a syndrome of inflammation and increased permeability, is associated with a constellation of clinical, radiological and physiological abnormalities that cannot be explained by, but may coexist with, left atrial or pulmonary capillary hypertension, and that the term ARDS should be reserved for the most severe end of this spectrum [3].

Several preoperative risk factors for ARDS have been identified, including age older than 60 years, male gender, chronic lung disease, reduced respiratory function test, prior radiation or chemotherapy, and concurrent cardiac disease. Perioperative risk factors include type and extent of lung resection, increased blood loss, blood transfusions, excessive volume of intraoperative fluids, and reoperation [4,5].

Studies that used the American-European consensus conference definitions for ARDS have reported an overall prevalence rate of 2.2 to 4.2% in patients who have undergone lung resection. The mortality rate from ARDS in these patients ranged from 52 to 65% [6,7]. Historically, the type of resection influences the mortality associated with ARDS; lower mortality rates are observed in patients undergoing lobar or sublobar resections, and higher rates are seen following pneumonectomy [8,9].

The purpose of our study was to describe the frequency associated with ARDS after lung resection in patients who required invasive mechanical ventilation (MV) in intensive care unit retrospectively. Additionally, we analyzed preoperative and perioperative factors that we hypothesized to be associated with ARDS.

## Materials and methods

All patients with ARDS developing after lung resection that required mechanical ventilation (MV) and admission to the intensive care unit (ICU) from January 2005 to February 2010, at Adnan Menderes University Medical Faculty Thoracic Surgery Department in Turkey were investigated in this retrospective study. ALI and ARDS were defined as per the American-European consensus conference [3].

All patients were evaluated by the same thoracic surgical team, and all preoperative studies were standardized. In addition to a history and physical examination, preoperative evaluation included chest radiography, pulmonary function testing, electrocardiography (ECG) and computerize tomography(CT) scans of the chest and upper abdomen. Quantitative ventilation/perfusion scanning, echocardiography, and positron emission tomography (PET) or brain imaging were performed to evaluate or extent of disease when appropriate.

Preoperative antimicrobial prophylaxis with cefazolin was administered routinely. After induction of anesthesia, a left or right double-tube lumen was introduced into the trachea, and their correct placements were confirmed by bronchoscopy before and after the patients were placed in the lateral position. During one lung ventilation (OLV), the lumen of the nonventilated side was left open to the air. All patients undergoing two lung ventilation (TLV) were ventilated with volume control ventilation with tidal volume (Vt) of 6 to 8 mL/kg, a respiratory rate to maintain PaCO_2 _between 35 and 40 mmHg, an inspiration-to-expiration ratio of 1:2. The plateau pressures values in all patients were below the range currently recommended as "a protective lung ventilation strategy" (below 30 cmH_2_O).

The demographic data for all patients who underwent lung resections included age, gender, diabetes mellitus, chronic alcoholism, smoking history, cardiovascular comorbidities (hypertension, coronary artery disease, heart failure, arrhythmia, or stroke), preoperative pulmonary function test results, American Society of Anesthesiologists (ASA) score and the indication for lung resection (benign or malign pathology). The classification of physical status by American Society of Anesthesiology (ASA) is a simple scoring system that correlates with surgical risk, ranging from ASA-I (no comorbidity, lowest risk) to ASA-V (unlikely to survive with or without surgery, highest risk). [ASA-I: Normally healthy patient, ASA-II: Patient with mild systemic disease, ASA-III: Patient with severe systemic disease that is not incapacitating, ASA-IV: Patient with an incapacitating systemic disease that is a constant threat to life, ASA-V: Moribund patient who is not expected to survive for 24 hours with or without operation] [10]. Patients were extubated at the end of the operation or shortly after arrival in the post anesthesia care unit, and were transferred to the surgical ward on the first postoperative day. Postoperative pain control was achieved with continuous IV or epidural patient-controlled analgesia. All lung resections (pneumonectomy, lobectomy, and sublobar resections) were performed through a standard posterolateral thoracotomy.

Type of pulmonary resection, duration of surgery and intraoperative fluid administration and blood products (erythrocyte suspension, fresh frozen plasma (FFP)) were also recorded.

If respiratory failure (ARDS) occurred at postoperative period, the patients were transferred to the ICU, where arterial blood gas analysis, ECG, and chest radiography were performed on admission and daily thereafter. Patients were ventilated with low-tidal-volume ventilation (6 to 8 mL per measured body weight), and positive end expiratory pressure levels ranged from 5 to 10 cm H_2_O (median, 7.5 cm H_2_O) (as a protective lung ventilation strategy).

## Statistical Analysis

Data are presented as median (range), absolute numbers, or percentages. Each of the preoperative and perioperative variables was examined using Fisher exact test for categorical variables and logistic regression for continuous variables. All statistical analyses were performed using statistical software (SPSS 11.0 Chicago, IL).

## Results

Over the course of five years (January 2005 to February 2010), 143 patients underwent lung resections at our institution [pneumonectomies, n = 10 (6.8%); lobectomies, n = 76 (51.7%); and sublobar wedge or segmentectomy resections, n = 57 (38.8%)]. Seventy-nine patients (53.7%) had malign pathology. Of the 143 patients, the median age was 56.77 years (range, 18-80 years). Demographic data and co-morbidities of all patients who underwent lung resection were shown in Table 1.

**Table 1:** Demographic data and pulmonary function test of all patients who underwent lung resection (n = 143)

Variables	Patients no and frequency (%)
**Age (older than 65 years)**	44 (29.9%)
**Gender Male**	89 (62.23%)
**Female**	54 (37.77%)
**Smoking history**	56 (39.16%)
**Alcohol abuse**	38 (25.9%)
**Cardiovascular co-morbidities**	47 (31.9%)
**Diabetes mellitus**	16 (10.9%)
**Chronic obstructive lung disease**	79 (53.7%)
**Indication for lung resection**	
**Malign Pathology**	79 (53.7%)
**Benign Pathology**	64 (46.3%)
**Anesthesia risk score**	
**ASA-I**	44 (29.9%)
**ASA-II**	91 (61.9%)
**ASA-III**	8 (5.4%)
**FEV1 less than 2 L**	59 (40.1%)
**Previous thoracic surgery**	22 (15%)
	

Of the 143 patients, 4 patients (2.7%) were died during hospitalization period. Eleven patients (7.5%) acquired ARDS requiring invasive MV and mortality ratio was 18.8% (2 patients) for these patients. The demographic data and comorbidities in the patients who acquired ARDS were summarized in Table 2.

**Table 2:** Demographic data of lung resections with acute lung injury

Variables	Patients no (n = 11) and frequency (%)
**Age (older than 65 years)**	3 (27.3%)
**Gender Male**	7 (63.63%)
**Female**	4 (36.36%)
**Smoking history**	5 (45%)
**Alcohol abuse**	9 (81.8%)
**Cardiovascular co-morbidities**	6 (54.6%)
**Diabetes mellitus**	3 (27.3%)
**Chronic obstructive lung disease**	8 (72.7%)
**Indication for lung resection**	
**Malign Pathology**	8 (72.7%)
**Benign Pathology**	3 (27.3%)
**Anesthesia risk score**	
**ASA-I**	1 (9.1%)
**ASA-II**	3 (27.3%)
**ASA-III**	7 (63.6%)
**FEV1 less than 2 L**	6 (54.5%)
**Previous thoracic surgery**	3 (27.3%)

Of the 11 ARDS patients, the median age was 62.09 years (range, 53-77 years). Six patients underwent pneumonectomy (right side, 5 patients; left side, 1 patient); 4 patients underwent lobectomy or bilobectomy; and 1 patient underwent sublobar resections. The mortality rate with ALI was highest after pneumonectomy (33.3%), followed by lobectomy (25%) and sublobar resections (0%).

Postoperative complications such as prolonged air leak, pneumothorax, empyema and wound infection were not significant (summarized in Table 3).

**Table 3:** Intraoperative transfusion requirement and postoperative complications (Patients no and frequency)

Variables	All patients [patients no or frequency (%)]	Patient with ALI
**Prolonged air leak**	4(2.1%)	0(0%)
**Pneumothorax**	12(6.2%)	0(0%)
**Empyema**	8(4.2%)	3(27.3%)
**Wound infection**	4(2.1%)	0(0%)
**Transfusion requirement** **Fresh frozen plasma (FFP)**	67(45.6%) 31(21.1%)	7(63.7%) 6(54.5%)
**Longer operation time (over 4 h)**	68(46.3%)	5(45.5%)
**Mortality**	4(2.1%)	2(18.18%)
		

Alcohol abuse [p = 0.01], ASA score [p = 0.001], FFP [p = 0.027] and pulmonary resection type [p = 0.032] were the factors found to be statistically significant for ARDS (Statistically values were summarized in Table 4).

**Table 4:** Preoperative, intraoperative and postoperative risk factors for ARDS

Variables	Odds ratio	Confidental interval	p value
**Alcohol abuse**	39.6	2.4-645.2	0.01
**ASA score**	1257.3	17.8-88604	0.001
**FFP**	28.6	1.4-562	0.027
**Pulmonary resection type**	1.4	1.2-1.9	0.032

Alcohol abuse, ASA score (the American Society of Anesthesiologists Physical Status classification score), FFP and pulmonary resection type were the factors found to be statistically significant for ARDS.

## Discussion

In the present study, postoperative ARDS due to lung resection performed in thoracic surgery patients was evaluated retrospectively. We observed that the predictive factors for ARDS were preoperative risk factors (such as alcohol abuse, higher ASA score classification), pulmonary resection type and the transfusion of fresh frozen plasma during intraoperative period.

The guidelines set out by the American-European Consensus Conference on ARDS have been widely adopted to describe post-thoracotomy ALI, previously coined postpneumonectomy pulmonary edema, low pressure edema or permeability pulmonary edema. Although the diagnosis of ALI/ARDS relies on specific criteria acute onset of hypoxemia, arterial oxygen pressure (PaO_2_)/fraction of inspired oxygen (FIO_2_) less than 300 for ALI and less than 200 for ARDS, diffuse radiological infiltrates and no evidence of elevated hydrostatic capillary pressure, a wide spectrum of lung injuries is encountered[3]. Importantly, two clinical patterns of post-thoracotomy ARDS should be distinguished corresponding to different pathogenic triggers: ARDS developing within 48-72 h after lung resection (primary ARDS) and a delayed form triggered by postoperative complications such as trachea-bronchial aspiration or pneumonia [8]. We examined primary ARDS in our study.

Our ARDS prevalence rate of 7.5% is higher than two studies of patients undergoing lung resection that acquired ARDS (as defined by the American-European consensus conference definitions) and required MV in literature [6,7]. However, our mortality rate of 27.3% for ARDS patients was lower than the 50% mortality rate reported in those studies [6,7]. We thought that, when we realized the symptoms of ARDS, we begun early MV therapy. It might be effective our mortality ratio.

ARDS was developed in six patients who underwent pneumonectomy; 4 patients who underwent lobectomy or bilobectomy; and 1 patient who underwent sublobar resections in our study. The mortality rate with ARDS was also highest after pneumonectomy (33.3%), followed by lobectomy (25%) and sublobar resections (0%). Similar to our results, the mortality from ARDS in previous reports was highest in patients who underwent a pneumonectomy as compared to those who underwent lesser resections [6-8,11-16]. It has been hypothesized that the larger volume of resected lung and greater reduction in lymphatic drainage may account for the higher mortality of ARDS after pneumonectomy [2].

Licker and colleagues reviewed 879 patients who underwent pulmonary resection and showed in multivariate analysis that excessive fluid administration, high intraoperative ventilatory pressures, pneumonectomy, and preoperative alcohol abuse were independent risk factors for ARDS [8].

In our study, all patients were ventilated with low-tidal-volume ventilation (6 to 8 mL per measured body weight), and positive end expiratory pressure levels ranged from 5 to 18 cm H_2_O (median, 7.5 cm H_2_O). Standard anesthesia induction and maintenance regimens, as well as intraoperative fluid restriction, were also used for all patients in our study. We ascribe our lower mortality rate in our study, in part, to our use of low-tidal-volume ventilation as a ventilatory management strategy in intraoperative and postoperative period. Licker and colleagues shows that both of high intraoperative ventilatory pressures and preoperative alcohol abuse were independent risk factors for ARDS [8]. In this respect, we detected that alcohol abuse was an independent risk factor for ARDS.

Actually, the identification of the correlation between alcohol abuse and ARDS after lung resection is new. It is not easy to directly link the two. Alcohol has been implicated in many other perioperative complications [8,17,18]. Furthermore, Boe and colleagues have identified alcohol abuse as an independent risk factor for the development of ARDS [19]. They claimed that alcohol abuse impairs immune function, decreases pulmonary antioxidant capacity, decreases alveolar epithelial cell function, alters activation of the renin angiotensin system, and impairs GM-CSF signaling [19].

The occurrence of ARDS is more frequently reported after those requiring multiple transfusions of fresh frozen plasma in lung resection [12,17]. The evidence of transfusion related ARDS (TRALI) is a clinical "experience" in which plasma known to contain strong anti-leukocytes, and particularly anti-monocyte antibodies, has caused severe lung damage in otherwise healthy individuals [19,20]. More recent in vitro experiments show that monocytes, anti-monocyte antibodies, and lung endothelium in co-culture can cause production of large amounts of cytokines and endothelial damage [21,22]. An alternative theory of TRALI pathogenesis suggests that abnormal lipids in cellular products cause neutrophil activation leading to lung damage [23]. Gajic and colleagues found an association with transfusion of FFP, but not with numbers of red cell units, or their age or leukocytes content [24]. Further studies confirmed an association between plasma transfusion and ARDS [25]. Moreover the results strongly suggested that female donor plasma was much more strongly associated with ARDS than male donor plasma, a finding that suggested a causal relationship rather than a simple association [26]. Leukocyte antibodies are found chiefly in females with a history of childbirth [25]. Similar to these reports, FFP transfusion has been found to be a predictor for the development of ARDS in our study.

Co-morbidity factors of the patients might have played a role in the development of the ARDS [7]. The classification of physical status and co-morbidity by the American Society of Anesthesiology (ASA) is a simple scoring system that correlates with surgical risk, ranging from ASA-I (no co-morbidity, lowest risk) to ASA-V (unlikely to survive with or without surgery, highest risk) [10]. Co-morbidities included diabetes mellitus, decreased preoperative pulmonary function test results and cardiovascular co morbidities (hypertension, coronary artery disease, heart failure, arrhythmia, or stroke). The ASA classification is used as a surrogate for the patient's underlying severity of illness and has been recommended for use in risk stratification in thoracic surgery [8,27]. We also suggest that ASA scores are independent risk factors for ARDS.

Although the patients in the general thoracic surgery are afflicted by multiple co morbid conditions, there are some confusing studies about the relationship of diabetes mellitus and ARDS in literature [28,29]. We observed that diabetes mellitus was not found to be a predictor for the development of ARDS in our study. Actually, Honiden and colleagues determined that clinical and experimental data indicate that diabetes is protective against the development of ARDS [29]. Independent of glycemic control, insulin has been shown to modulate inflammation [29]. More research is required to understand the role of diabetes, insulin, and hyperglycemia in critically ill patients with ALI.

We concluded that in patients who underwent lung resection, preoperative risk factors (such as alcohol abuse, higher ASA score classification), pulmonary resection type and the transfusion of FFP during intraoperative period were the predictors of development of ARDS.

## Competing interests

The authors declare that they have no competing interests.

## Authors' contributions

All authors read and approved the final manuscript.
